# Global lung function initiative 2012 reference values for spirometry in Asian Americans

**DOI:** 10.1186/s12890-018-0658-9

**Published:** 2018-05-31

**Authors:** Jingzhou Zhang, Xiao Hu, Xinlun Tian, Kai-Feng Xu

**Affiliations:** 1 Department of Respiratory Medicine, Peking Union Medical College Hospital, Peking Union Medical College & Chinese Academy of Medical Sciences, Beijing, 100730 China; 2 Department of Epidemiology, Mailman School of Public Health, Columbia University, New York, NY USA; 3 Yale School of Public Health, Yale University, New Haven, CT USA

**Keywords:** Asian Americans, Lung function, LLN, Spirometry, Z-score

## Abstract

**Background:**

Spirometry reference values specifically designed for Asian Americans are currently unavailable. The performance of Global Lung Function Initiative 2012 (GLI-2012) equations on assessing spirometry in Asian Americans has not been evaluated. This study aimed to assess the fitness of relevant GLI-2012 equations for spirometry in Asian Americans.

**Methods:**

Asian subjects who never smoked and had qualified spirometry data were extracted from the National Health and Nutrition Examination Survey (NHANES) 2011–2012. Z-scores of forced expiratory volume in 1 s (FEV_1_), forced vital capacity (FVC), and FEV_1_/FVC were separately constructed with GLI-2012 equations for North East (NE) Asians, South East (SE) Asians, and individuals of mixed ethnic origin (Mixed). In addition, Proportions of subjects with observed spirometry data below the lower limit of normal (LLN) were also evaluated on each GLI-2012 equation of interest.

**Results:**

This study included 567 subjects (250 men and 317 women) aged 6–79 years. Spirometry z-scores (z-FEV_1_, z-FVC, and z-FEV_1_/FVC) based on GLI-2012 Mixed equations had mean values close to zero (− 0.278 to − 0.057) and standard deviations close to one (1.001 to 1.128); additionally, 6.0% (95% confidence interval (CI) 3.1–8.9%) and 6.4% (95% CI 3.7–9.1%) of subjects were with observed data below LLN for FEV_1_/FVC in men and women, respectively. In contrast, for NE Asian equations, all mean values of z-FEV_1_ and z-FVC were smaller than − 0.5; for SE Asian equations, mean values of z-FEV_1_/FVC were significantly smaller than zero in men (− 0.333) and women (− 0.440).

**Conclusions:**

GLI-2012 equations for individuals of mixed ethnic origin adequately fitted spirometry data in this sample of Asian Americans. Future studies with larger sample sizes are needed to confirm these findings.

**Electronic supplementary material:**

The online version of this article (10.1186/s12890-018-0658-9) contains supplementary material, which is available to authorized users.

## Background

Accurate interpretation of pulmonary function test results, which requires valid spirometry reference values, is of material importance to respiratory medicine. In addition to gender, age, and height, race/ethnicity acts as another major determinant of lung function [1–3]. Therefore, it is recommended that spirometry reference values established with healthy people of similar race/ethnicity be applied to a certain population whenever possible. The European Respiratory Society (ERS)/American Thoracic Society (ATS) recommended spirometry reference values that were based on a sample from the third National Health and Nutrition Examination Survey (NHANES III) for population aged 8–80 years in US [4, 5]. Nonetheless, limited by race/ethnicity classification in NHANES III, spirometry reference values for Asian Americans were unable to be produced through Hankinson et al.’s study [5].

Previous studies showed that Asian Americans had clinically significantly lower forced expiratory volume in 1 s (FEV_1_) and forced vital capacity (FVC) compared with Caucasian people in US [6–11]. Accordingly, a correction factor for FEV_1_ and FVC has been developed and calibrated to be applied to NHANES III Caucasian equations when assessing spirometry in Asian Americans. Specifically, 0.94 and 0.88 have been sequentially proposed as the correction factor for FEV_1_ and FVC [4, 12, 13]. A recent systemic review suggested that a correction factor of 0.88 was more suitable than 0.94 to be applied to NHANES III Caucasian reference values for FEV_1_ and FVC evaluation in Asian Americans [14].

In 2012, the Global Lung Function Initiative (GLI-2012) published all-age-covering spirometry predictive equations for multiple ethnicities, including North East (NE) Asian and South East (SE) Asian [15]. In addition, a set of GLI-2012 equations were designed for individuals of mixed ethnic origin (Mixed) [15]. Although with mixed results, GLI-2012 equations showed clinically acceptable generalisability to spirometry in several validation samples [16–21]. Therefore, relevant GLI-2012 equations are potentially useful for evaluating lung function of Asian Americans. Nonetheless, performance of GLI-2012 reference equations on assessing spirometry in Asian Americans has not been evaluated.

Asian people, including Asian alone and in combination with other races, account for more than 17.3 million (5.6%) of total American population in 2010 [22]. Of note, the total US Asian population increased by 5.4 million (45.6%) from 2000 to 2010, and is projected to grow to 48.6 million by 2060 [23, 24]. Owing to the remarkable quantity and rapid growth of Asian population in US, it is clinically important to assess spirometry reference values that have been recommended for or can be potentially used in that population. Herein, we conducted this study to assess the fitness of relevant GLI-2012 equations and NHANES III reference values for spirometry in Asian Americans.

## Methods

### Study design

Asian subjects from NHANES 2011–2012, where spirometry data were available, were included in this study. The NHANES utilized a complex, multistage, probability sampling design to collect health and nutrition data from a nationally representative sample of civilian, non-institutionalized people in US each year. Since the year of 2011, NHANES has started to oversample Asian population in US and code them as “non-Hispanic Asian” for race/ethnicity, which provided opportunity for investigating health conditions specifically on Asian Americans [25]. NHANES 2011–2012 finally released demographic, nutritional, and health data of 1282 non-Hispanic Asian participants, which served as the basis for this study. NHANES protocols were reviewed and approved by the Research Ethics Review Board of National Center for Health Statistics, and written informed consent was obtained from each NHANES participant.

**Fig. 1:**
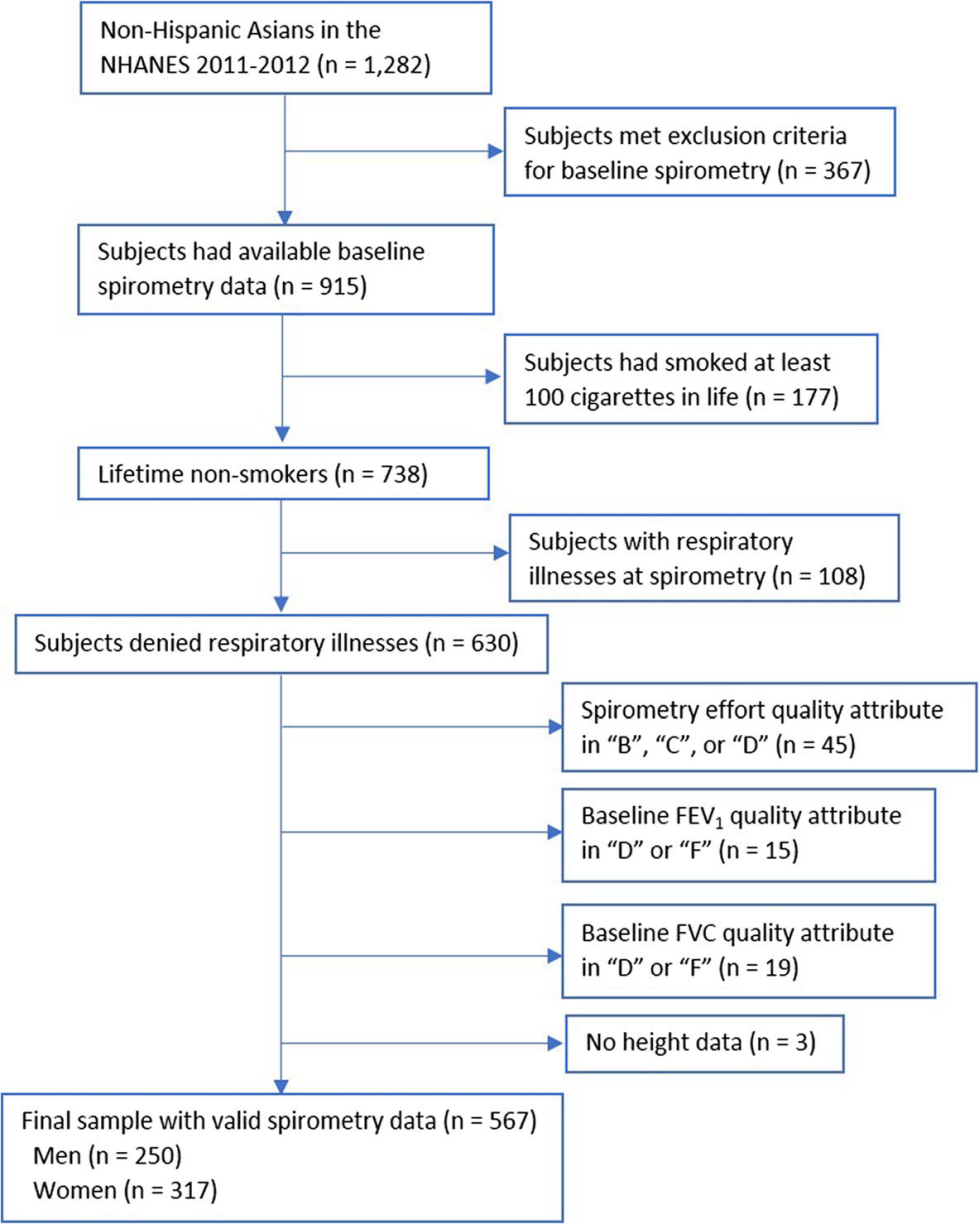
Flowchart of study sample selection

This study’s exclusion criteria were: 1) examinees who did not qualify for a baseline spirometry test; 2) current or past smokers (defined as those who had smoked at least 100 cigarettes in life); 3) participants who reported respiratory illnesses (cough, cold, phlegm, runny nose, or other respiratory illnesses) seven days prior to the examination; 4) baseline spirometry effort quality attribute of “B”, “C” or “D”, or baseline FEV_1_ or FVC quality attribute of “D (questionable results, use with caution)” or “F (results not valid)” [26, 27]. A detailed study sample inclusion and exclusion process is shown in Fig. 1.

### Spirometry measurements

Participants aged 6–79 years were eligible for spirometry tests in NHANES 2011–2012. Examinees who had breathing problem requiring oxygen/taking deep breath, current ear infection, eye/chest/abdominal surgery, or stroke/heart attack in the past three months, tuberculosis in the past year, or coughing up blood in the past month were excluded from a baseline spirometry. Technicians received formal training and used an Ohio 822/827 dry-rolling seal volume spirometer (Ohio Medical, Gurnee, IL, USA) for spirometry tests. Regular calibration of spirometry equipment and rigorous spirometry curves quality control were conducted by health technicians and were subsequently verified by supervisory staff [28].

### Statistical analysis

The fitness of GLI-2012 reference equations designed for NE Asians, SE Asians, and individuals of mixed ethnic origin and Caucasians were evaluated for spirometry in this sample. GLI-2012 equations were designed using the “Generalized Additive Models for Location, Scale, and Shape (GAMLSS)” method, which permitted the fitness of mean (M), coefficient of variance (S), and skewness (L) of spirometry data [15, 29]. Z-scores of FEV_1_ (z-FEV_1_), FVC (z-FVC), and FEV_1_/FVC (z-FEV_1_/FVC) were calculated using the formula: z-score = ((observed value/M) ^ L - 1) / (L*S). The z-score is defined as how many standard deviations (SDs) a measured value is from predicted value (z-score = (observed - predicted)/SD). One may argue that the z-score is a more appropriate approach to reporting lung function data than using % predicted by considering lung function related variables (age, height, ethnicity, etc.) [30]. The proportion of subjects with observed spirometry data below lower limit of normal (LLN), which corresponds to the 5th percentile of predicted values, were also evaluated for FEV_1_, FVC, and FEV_1_/FVC on each GLI-2012 equation of interest. The cutoff z-score of LLN was calculated with the formula: LLN z-score = − 1.6445 * (SD of z-scores).

Student’s t-tests were used to examine the difference between the mean of z-scores and zero. Bland-Altman plots of spirometry predictions based on NHANES III Caucasian equations with 0.88 as the correction factor for FEV_1_ and FVC against GLI-2012 Mixed equations were generated (difference = NHANES III prediction – GLI-2012 prediction). Bland-Altman plots are used to describe agreement between two quantitative methods of measurement by calculating the mean difference and 95% limits of agreement (1.96*SD of the difference) between the two measurements [31]. A two-sided *P* < 0.05 was considered statistically significant for all tests. Data analyses were performed with SAS 9.4 (SAS Institute, Cary, NC, USA) and R version 3.4.0 (R Foundation for Statistical Computing, Vienna, Austria).

## Results

### Sample characteristics (Table 1)

**Fig. 2:**
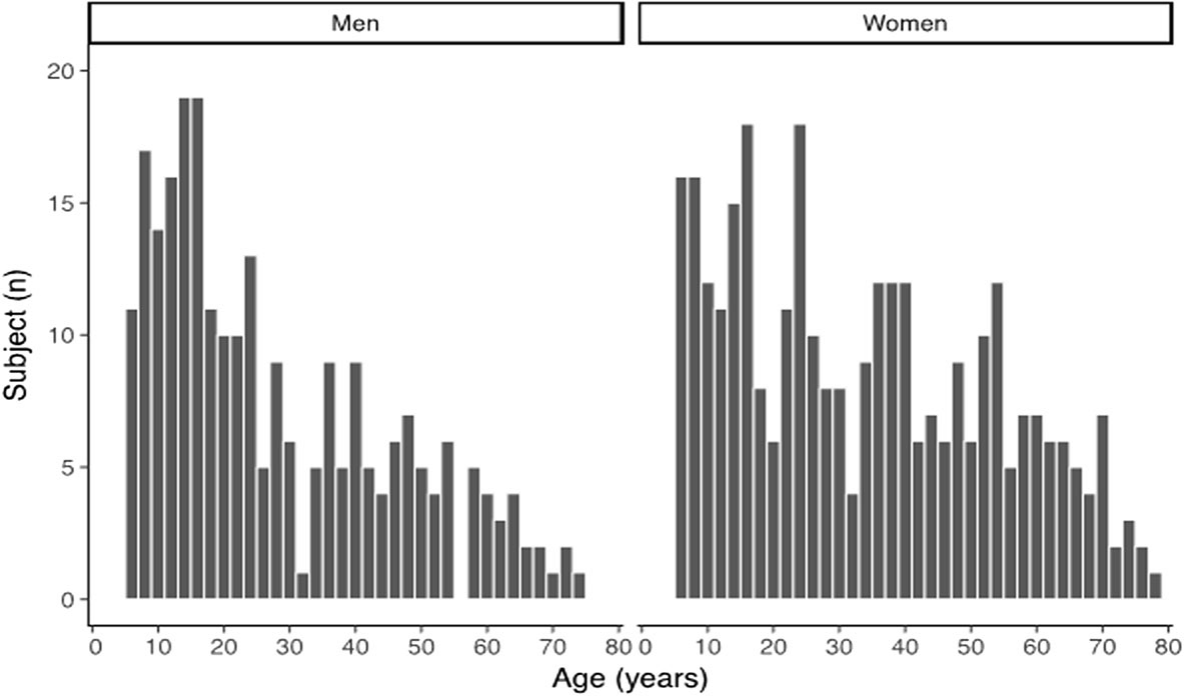
Age distribution of study subjects by gender

Five hundred and sixty-nine Asian participants (250 men and 317 women) were finally included in this analysis. The mean (SD) age were 28.4 (17.8) years for men and 34.3 (19.7) years for women; and the age range for men and women were 6 to 75 years and 6 to 79 years, respectively (Fig. 2). The mean (SD) height for men and women were 164.1 (15.6) cm and 154.2 (11.4) cm, respectively. In this sample, there were 17 (6.8%) men and 19 (6.0%) women who had a BMI ≥ 30 kg/m^2^. Additionally, 38.4% of men and 31.6% of women were born in US. Among those who were not born in US, 31.6% of men and 37.3% of women had lived in US for more than 20 years, whereas 27.0% of men and 20.6% of women had been in US for less than 5 years.

**Table 1:** Baseline characteristics of sample subjects by gender ^a^

Characteristics	Gender	
	Men (*n* = 250)	Women (*n* = 317)
Age (year)	28.4 ± 17.8	34.3 ± 19.7
	23 (14, 41)	32 (16, 51)
Height (cm)	164.1 ± 15.6	154.2 ± 11.4
Weight (kg)	62.8 ± 19.4	53.9 ± 14.4
BMI (kg/m^2^)	22.7 ± 4.7	22.3 ± 4.6
Born in the U.S.	96 (38.4%)	100 (31.6%)
Length of time in U.S. ^b^		
Less than 5 years	41 (27.0%)	44 (20.6%)
5 to 10 years	28 (18.4%)	26 (12.2%)
10 to 20 years	35 (23.0%)	64 (29.9%)
More than 20 years	48 (31.6%)	80 (37.3%)
FEV_1_ (L)	3.159 ± 0.904	2.344 ± 0.634
FVC (L) ^c^	3.761 ± 1.052	2.785 ± 0.710
FEV_1_/FVC ^c^	0.84 ± 0.07	0.84 ± 0.08

*BMI* body mass index, *FEV*_*1*_ forced expiratory volume in 1 s, *FVC* forced vital capacity

^a^data were presented as mean ± standard deviation, median (interquartile range), or as number (percentage)

^b^for participants who were not born in the United States; 2 missing these data for men and 3 missing these data for women

^c^1 missing these data for men and 6 missing these data for women

### Performance of GLI-2012 equations (Table 2)

**Fig. 3:**
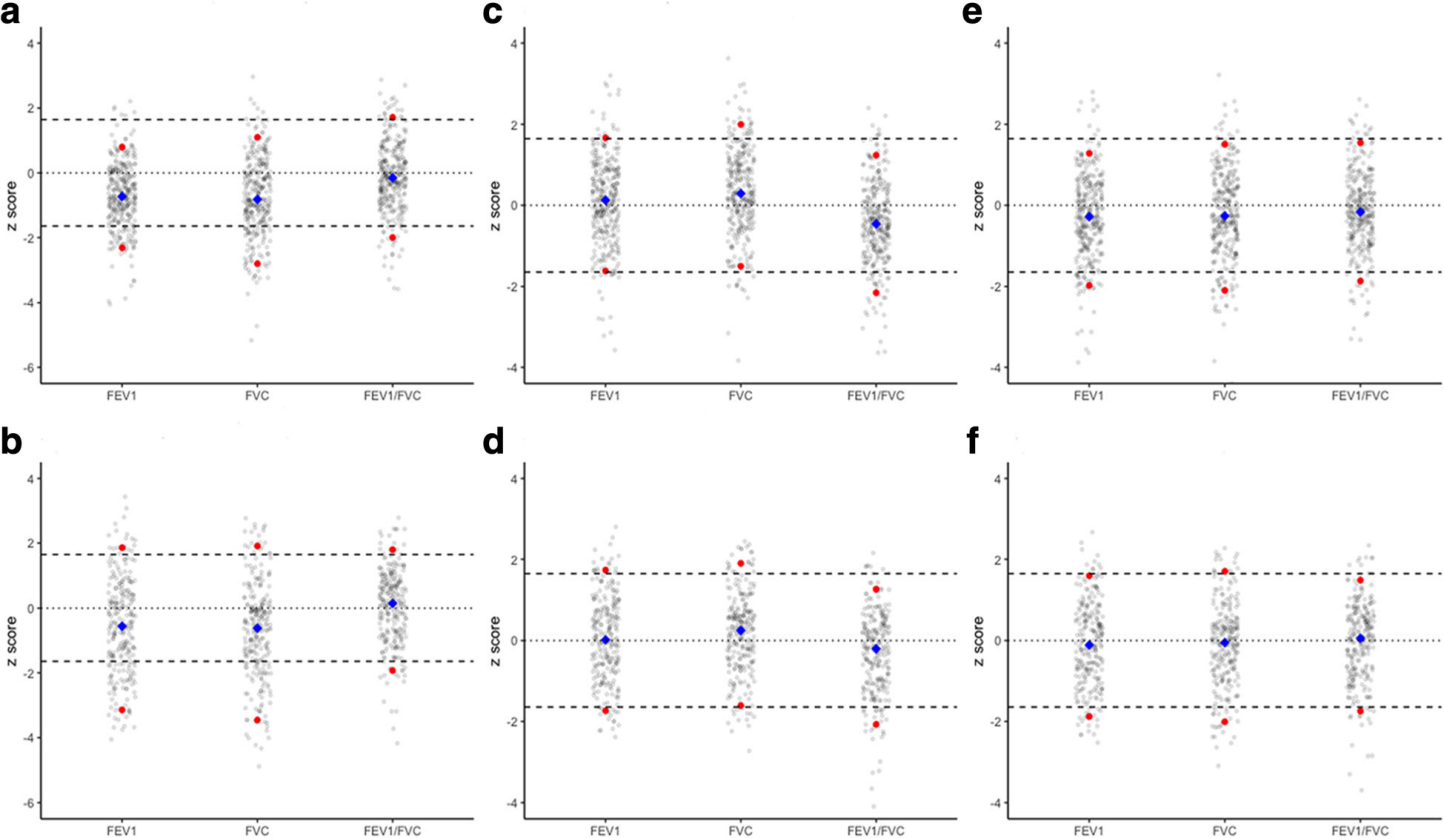
Distributions of z-scores of FEV_1_, FVC, and FEV_1_/FVC based on GLI-2012 equations for North East Asians, South East Asians, and individuals of mixed ethnic origin. Panels A and B showed z-score distributions based on GLI-2012 equations for North East Asians in women and men, respectively; panels C and D showed z-score distributions based on GLI-2012 equations for South East Asians in women and men, respectively; and panels E and F showed z-score distributions based on GLI-2012 equations for individuals of mixed ethnic origin in women and men, respectively. In this graph, red dot denotes 5th and 95th percentiles of observed spirometry data; blue diamond denotes median of observed values; solid line represents a z-score of zero; and dotted line represents z-scores of ±1.96. *FEV*_*1*_*: forced expiratory volume in 1 s; FVC: forced vital capacity; GLI: Global Lung Function Initiative*

For NE Asian equations, all mean (median) values of z-FEV_1_ and z-FVC were smaller than − 0.5 in both men and women, with the lowest as − 0.743 (− 0.819) for z-FVC in women. For SE Asian equations, mean values of z-FEV_1_/FVC were − 0.333 in men and − 0.440 in women, all significantly different from zero. In terms of the Mixed equations, all mean values of z-FEV_1_, z-FVC, and z-FEV_1_/FVC were not significantly different from zero in men; and in women, although statistically significantly different from zero, all absolute differences were within 0.3. SDs of z-scores based on GLI-2012 SE Asian equations and the Mixed equations ranged from 1.002 to 1.089 and 1.001 to 1.128, respectively, indicating that those equations adequately fitted variations of our spirometry data. In contrast, SDs of z-FEV_1_ and z-FVC based on GLI-2012 NE Asian equations were 1.512 and 1.517, respectively. Distributions of z-scores based on GLI-2012 equations were showed in Fig. 3. For Caucasian equations, mean values of z-FEV_1_ and z-FVC were substantially smaller than zero in both men and women (Additional file 1: Fig. S1). Also, plots of spirometry z-scores for GLI-2012 reference eqs. (NE, SE, and the Mixed) against age in men and women were showed in Additional file 2: Fig. S2 and Additional file 3: Fig. S3, respectively.

**Table 2:** Spirometry z-scores of the present study population based on the GLI-2012 equations for North East Asians, South East Asians, and individuals of mixed ethnic origin

GLI-2012 equations	Statistics	z-FEV_1_		z-FVC		z-FEV_1_/FVC	
		Men	Women	Men	Women	Men	Women
North East Asians	Mean ± SD	−0.571 ± 1.512	− 0.703 ± 1.029	−0.695 ± 1.517	−0.743 ± 1.223	0.021 ± 1.177	−0.135 ± 1.089
	95% CI of mean	(−0.759, − 0.382) *	(−0.817, − 0.589) *	(−0.884, − 0.506) *	(−0.880, − 0.607) *	(−0.124, 0.167)	(− 0.257, − 0.014) *
	Median	−0.558	− 0.730	−0.619	− 0.819	0.148	− 0.152
	(5th, 95th percentile)	(−3.143, 1.855)	(−2.313, 0.799)	(− 3.458, 1.909)	(− 2.797, 1.099)	(− 1.928, 1.798)	(− 1.999, 1.716)
	N (%) < LLN	28 (11.2%)	51 (16.1%)	33 (13.3%)	42 (13.5%)	12 (4.8%)	20 (6.4%)
	95% CI of % < LLN	(7.3, 15.1%)	(12.1, 20.1%)	(9.1, 17.5%)	(9.7, 17.3%)	(2.1, 7.5%)	(3.7, 9.1%)
South East Asians	Mean ± SD	−0.037 ± 1.052	0.105 ± 1.080	0.177 ± 1.002	0.312 ± 1.089	−0.333 ± 1.055	−0.440 ± 1.010
	95% CI of mean	(−0.094, 0.168)	(− 0.014, 0.225)	(0.051, 0.302) *	(0.190, 0.433) *	(−0.465, − 0.201) *	(−0.552, − 0.327) *
	Median	0.017	0.126	0.251	0.290	−0.203	−0.462
	(5th, 95th percentile)	(−1.736, 1.733)	(−1.619, 1.667)	(− 1.610, 1.899)	(− 1.502, 1.994)	(− 2.073, 1.264)	(− 2.154, 1.239)
	N (%) < LLN	13 (5.2%)	8 (2.5%)	12 (4.8%)	10 (3.2%)	23 (9.2%)	31 (10.0%)
	95% CI of % < LLN	(2.4, 8.0%)	(0.8, 4.2%)	(2.1, 7.5%)	(1.2, 5.2%)	(5.6, 12.8%)	(6.7, 13.3%)
Individuals of mixed ethnic origin	Mean ± SD	−0.101 ± 1.051	− 0.278 ± 1.079	−0.112 ± 1.050	−0.212 ± 1.128	−0.057 ± 1.020	−0.152 ± 1.001
	95% CI of mean	(−0.232, 0.030)	(− 0.397, − 0.159) *	(−0.229, 0.033)	(− 0.338, − 0.086) *	(−0.185, 0.070)	(− 0.264, − 0.040) *
	Median	−0.111	− 0.284	−0.051	− 0.266	0.055	− 0.167
	(5th, 95th percentile)	(−1.881, 1.591)	(−1.975, 1.282)	(−2.007, 1.702)	(− 2.096, 1.508)	(− 1.745, 1.484)	(− 1.865, 1.547)
	N (%) < LLN	18 (7.2%)	20 (6.3%)	20 (8.0%)	25 (8.0%)	15 (6.0%)	20 (6.4%)
	95% CI of % < LLN	(4.0, 10.4%)	(3.6, 9.0%)	(4.6, 11.4%)	(5.0, 11.0%)	(3.1, 8.9%)	(3.7, 9.1%)

*GLI* Global Lung Function Initiative, *SD* standard deviation, *CI* confidence interval, *FEV*_*1*_ forced expiratory volume in 1 s, *FVC* forced vital capacity, *z-FEV*_*1*_ FEV_1_ z-score, *z-FVC* FVC z-score, *z-FEV*_*1*_*/FVC* FEV_1_/FVC z-score, *LLN* lower limit of normal

*P < 0.05 for student’s t-tests comparing mean values of z-scores and zero

Regarding proportion of observed spirometry data below LLN (% < LLN), the Mixed equations showed a satisfactory overall performance. Specifically, 6.0% (95% confidence interval (CI): 3.1–8.9%) and 6.4% (95% CI: 3.7–9.1%) of z-FEV_1_/FVC were below LLN for men and women, respectively. In contrast, according to SE Asian equations, 9.2% (95% CI: 5.6–12.8%) of z-FEV_1_/FVC in men and 10.0% (95% CI: 6.7–13.3%) of z-FEV_1_/FVC in women were below LLN; for NE Asian equations, all % < LLN for z-FEV_1_ and z-FVC were significantly larger than 5% (11.2 to 16.2%).

In addition, we confirmed that the NHANES III Caucasian reference values with a correction factor of 0.88 for FEV_1_ and FVC satisfactorily fitted the spirometry data (FEV_1_ and FVC) of this sample (data not shown).

### Agreement between NHANES III and GLI-2012 predictions

**Fig. 4:**
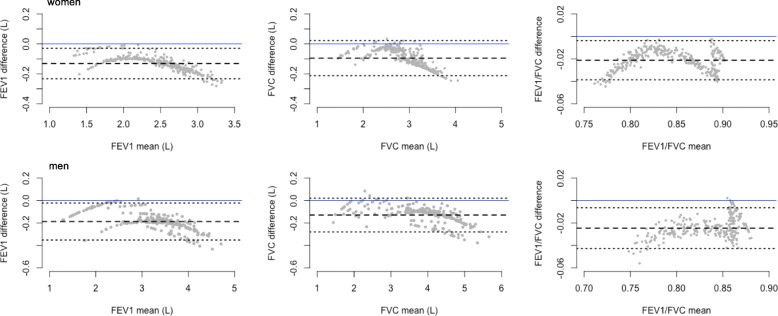
Bland-Altman plots of spirometry predictions using NHANES III Caucasian values with a correction factor of 0.88 for FEV_1_ and FVC against those with GLI-2012 equations for individuals of mixed ethnic origin (difference = NHANES III prediction – GLI-2012 prediction). In this graph, dashed line represents the mean difference; dotted line represents 95% confidence interval of the mean difference; solid line represents the value of zero. *NHANES III: The Third National Health and Nutrition Examination Survey; FEV*_*1*_*: forced expiratory volume in 1 s; FVC: forced vital capacity; GLI: Global Lung Function Initiative*

Overall, lung function predictions based on NHANES III Caucasian reference values with a correction factor of 0.88 for FEV_1_ and FVC were smaller than those based on the GLI-2012 equations for FEV_1_, FVC, and FEV_1_/FVC (Fig. 4). The average differences in FEV_1_ (L), FVC (L), and FEV_1_/FVC (%) predictions were − 0.187, − 0.130, and − 2.46 for men, and − 0.131, − 0.095, and − 2.12 for women, respectively.

## Discussion

In this population-based cross-sectional analysis of lung function, we were the first to assess the generalisability of relevant GLI-2012 reference equations to spirometry in Asian Americans. In addition, we evaluated the agreement of lung function predictions between the NHANES III Caucasian values with a correction factor of 0.88 for FEV_1_ and FVC and the GLI-2012 equations for individuals of mixed ethnic origin.

Our findings showed that GLI-2012 Mixed equations adequately fitted FEV_1_, FVC, and FEV_1_/FVC data of our sample for both gender. GLI-2012 Mixed equations were designed for people of mixed ethnic origin, which we believe current Asian Americans could be categorized into due to the following several reasons. First, in the year 2010, around 16% of Asian Americans were Asian in combination with one or more other races, among whom Asian in combination with White were the majority [22]. Second, US Asian population consists of more than twenty subgroups, with Chinese, Indian, Filipino, Vietnamese, Korean, and Japanese accounting for the most in quantity [22]. Third, due to diversities of birth country and years living in US, which is readily translated into difference in environmental exposures and socioeconomic status, Asian Americans may have quite different lung function development [32–37]. Therefore, Asian Americans are genetically, environmentally, and socioeconomically heterogeneous in nature, which may explain the satisfactory performance of GLI-2012 Mixed equations in fitting spirometry data in this sample.

GLI-2012 NE Asian equations were built based on two datasets, one collected from North China and the other from South Korea; whereas the GLI-2012 SE Asian equations were derived from a collated dataset consisting of five subsamples from South Asia and a subsample from US [15]. Quanjer et al. found that the two subsamples of NE Asians had significantly larger lung function than the six subsamples of SE Asians, and therefore they constructed spirometry predictive equations separately for NE Asians and SE Asians [15]. Not surprisingly, GLI-2012 NE Asian equations led to substantially larger FEV_1_ and FVC predictions compared with observed data in our sample for both gender, strongly suggesting against the application of those equations to assessing spirometry in Asian Americans. GLI-2012 SE Asian equations, while performed satisfactorily in fitting FEV_1_ and FVC, contributed to significantly larger FEV_1_/FVC predictions compared with the observed data, which will potentially result in an overdiagnosis of chronic obstructive pulmonary disease in Asian Americans.

Generally, both the GLI-2012 Mixed equations and the NHANES III Caucasian reference values with a correction factor of 0.88 adequately fitted the lung function data in this sample. However, GLI-2012 equations possess several potential advantages over the NHANES III reference values. First, as all-age-covering spirometry reference values, GLI-2012 equations are valid for people aged 3 to 95 years old [38]; the NHANES III equations, in contrast, have a comparably narrower valid age range of 8 to 80 years. Of note, in this study we were not able to evaluate the fitness of GLI-2012 equations for spirometry in Asian Americans aged outside 6 to 79 years. Secondly, GLI-2012 equations were designed with a semiparametric predictive modelling method, which was able to fit variance and skewness of spirometry data in addition to the mean value [39]. Moreover, splines used in GLI-2012 equations modeled age-related variations for spirometry data. NHANES III equations were built based on quadratic function for FEV_1_ and FVC and linear function for FEV_1_/FVC. Thus, compared with GLI-2012 equations, NHANES III equations were less likely to reflect actual patterns of spirometry data due to their fixed function formats. Thirdly, NHANES III equations for FEV_1_/FVC LLN and equations for FEV_1_/FVC are same as each other except different intercepts. Therefore, according to NHANES III equations, LLN for FEV_1_/FVC differs from FEV_1_/FVC by a constant magnitude regardless of a subject’s age. However, since LLN theoretically corresponds to the 5th percentile of spirometry data and lung function varies with age, it is conceptually insufficient to define LLN as a constant difference to the mean for the entire age range. GLI-2012 reference values address this issue by defining LLN with spirometry z-scores, a way comprehensively taking mean, variance, and skewness of spirometry data into consideration.

The GLI-2012 equations have been proposed to be adopted worldwide in order to standardise the interpretation of lung function [40]. Admittedly, the application of a correction factor to the NHANES III Caucasian reference values offers a practical solution to assessing spirometry in Asian Americans. However, the rationale behind the development of a correction factor, which is only for temporary use, is not conceptually and methodologically ideal. Based on the current findings and what has been discussed above, it is reasonable to regard GLI-2012 Mixed equations as superior to the NHANES III Caucasian reference values with a correction factor for evaluating spirometry in Asian Americans. In particular, the ready availability of spirometry z-scores and LLN from the GLI-2012 equations could possibly provide a convenient approach to the diagnosis and severity stratification of obstructive lung diseases. Therefore, with the rapid increase of Asian population in US, the application of GLI-2012 Mixed equations to Asian Americans is clinically important.

This study has several limitations. First, the sample size of this study is relatively small. However, we would argue that our sample sizes of men and women are both large enough for validating spirometry reference values, which requires at least 150 subjects for each gender [41]. Second, as shown in Fig. 2, the distributions of age are right skewed in both men and women. Especially for men, the proportion of adults and elderly people is relatively small, which may limit the power of this study in that population. This issue is clinically relevant in that obstructive lung diseases, where lung function references are widely used, are most prevalent in elderly people. As the accrual of NHANES data of Asian Americans, the fitness of GLI-2012 equations could be better evaluated in the near future.

## Conclusions

In this cross-sectional analysis of lung function from a nationally representative sample of US Asian population, we showed that the GLI-2012 reference equations for individuals of mixed ethnic origin performed adequately on fitting spirometry data of this sample. Considering the strengths of GLI-2012 equations such as all-age-covering capacity and readily z-score calculation and LLN definition, the GLI-2012 equations for individuals of mixed ethnic origin are reasonably considered as a useful set of tools in evaluating spirometry in Asian Americans. Further studies with larger sample sizes covering wider age ranges, especially the most elderly (> 80 years) people, are warranted to confirm these findings.

## Additional files

Additional file 1: **Figure S1.** Distributions of z-scores of FEV_1_, FVC, and FEV_1_/FVC based on GLI-2012 equations for Caucasians. (PDF 63 kb)

Additional file 2: **Figure S2.** Distributions of z-FEV_1_, z-FVC, and z-FEV_1_/FVC based on GLI-2012 equations for NE Asians, SE Asians, and individuals of mixed ethnic origin against age in men. (PDF 185 kb)

Additional file 3: **Figure S3.** Distributions of z-FEV_1_, z-FVC, and z-FEV_1_/FVC based on GLI-2012 equations for NE Asians, SE Asians, and individuals of mixed ethnic origin against age in women. (PDF 208 kb)

## Abbreviations

ATS: American Thoracic Society
ERS: European Respiratory Society
FEV_1_: Forced Expiratory Volume in 1 s
FVC: Forced Vital Capacity
GLI: Global Lung Function Initiative
LLN: Lower Limit of Normal
NHANES: National Health and Nutrition Examination Survey
North East Asian: NE Asian
South East Asian: SE Asian
