# Supplementary figures and images for: Global lung function initiative 2012 reference values for spirometry in Asian Americans
Source: BMC Pulm Med. 2018 May 31;18:95. doi: 10.1186/s12890-018-0658-9 (PMC5984415; doi:10.1186/s12890-018-0658-9)

Equations for Caucasian (Male)

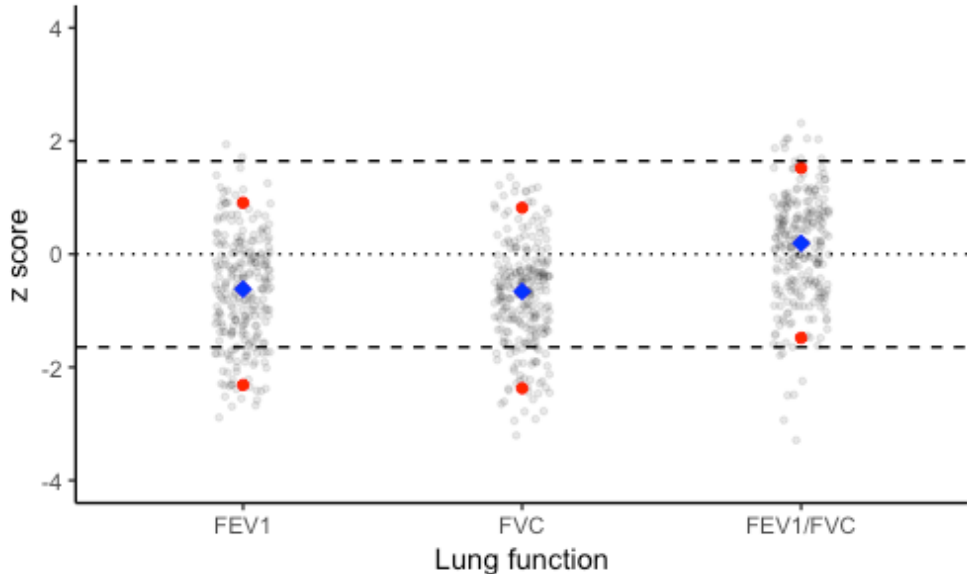

Equations for Caucasian (Female)

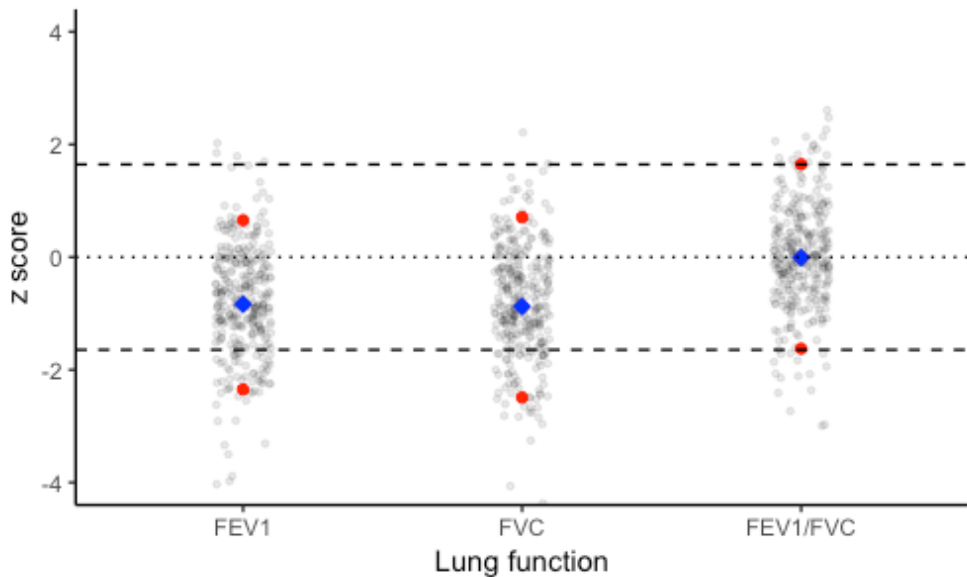

Supplement: Supplementary file 1 — Figure S1. Distributions of z-scores of FEV1, FVC, and FEV1/FVC based on GLI-2012 equations for Caucasians. (PDF 63 kb) [file 12890_2018_658_MOESM1_ESM.pdf]
