# Supplementary material for: Global lung function initiative 2012 reference values for spirometry in Asian Americans
Source: BMC Pulm Med. 2018 May 31;18:95. doi: 10.1186/s12890-018-0658-9 (PMC5984415; doi:10.1186/s12890-018-0658-9)
Supplement: Supplementary file 3 — Figure S3. Distributions of z-FEV1, z-FVC, and z-FEV1/FVC based on GLI-2012 equations for NE Asians, SE Asians, and individuals of mixed ethnic origin against age in women. (PDF 208 kb) [file 12890_2018_658_MOESM3_ESM.pdf]

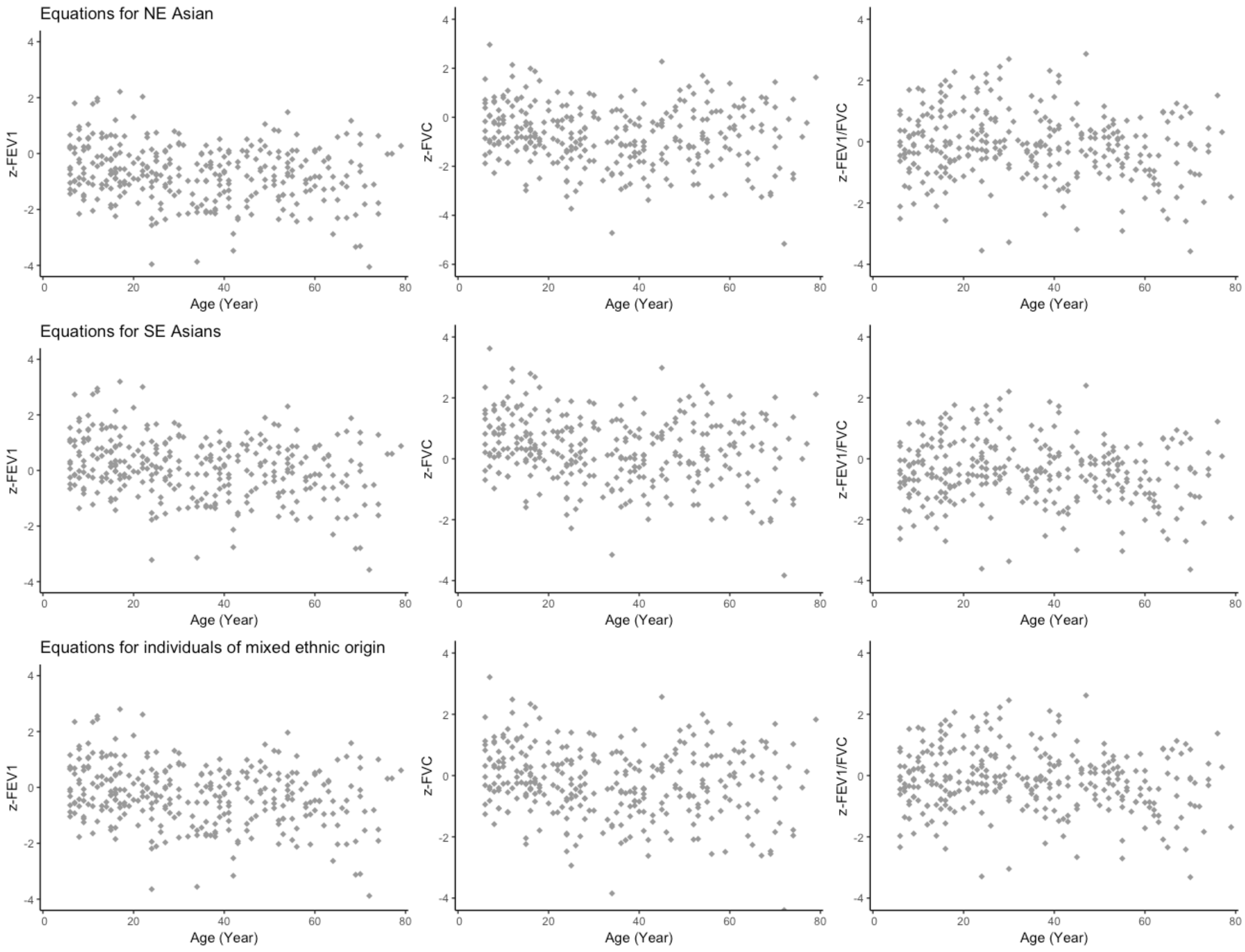
Equations for NE Asian
Equations for SE Asians
Equations for individuals of mixed ethnic origin
z-FEV1
z-FVC
z-FEV1/FVC
Age (Year)
